# Biocompatibility Study of Purified and Low-Temperature-Sterilized Injectable Collagen for Soft Tissue Repair: Intramuscular Implantation in Rats

**DOI:** 10.3390/gels10100619

**Published:** 2024-09-26

**Authors:** Tae-Hoon Koo, Jason K. Lee, Shawn P. Grogan, Ho Jong Ra, Darryl D. D’Lima

**Affiliations:** 1 D.med LLC, 111, Sagimakgol-ro, Jungwon-gu, Seongnam-si 13202, Gyeonggi-do, Republic of Korea; glenkoo@dmed.co.kr; 2 Shiley Center for Orthopaedic Research and Education at Scripps Clinic, 10666 N Torrey Pines Road, MS126, La Jolla, CA 92037, USA; sgrogan@scripps.edu (S.P.G.)

**Keywords:** collagen, injectable, skin, purification, sterilization, biocompatibility, implantation

## Abstract

The clinical application of collagen-based biomaterials is expanding rapidly, especially in tissue engineering and cosmetics. While oral supplements and injectable skin boosters are popular for enhancing skin health, clinical evidence supporting their effectiveness remains limited. Injectable products show potential in revitalizing skin, but safety concerns persist due to challenges in sterilization and the risk of biological contamination. Traditional methods of sterilization (heat and irradiation) can denature collagen. This study addresses these issues by introducing a novel technique: the double filtration and low-temperature steam sterilization of a collagen gel. In vitro tests documented the sterility and confirmed that the collagen did not show cytotoxicity, degradation, integrity, and viscosity characteristics changes after the processing and sterilization. The collagen gel induced new collagen expression and the proliferation of human dermal fibroblasts when the cells were cultured with the collagen gel. An in vivo study found no adverse effects in rats or significant lesions at the implantation site over 13 weeks. These results suggest that this novel method to process collagen gels is a safe and effective skin booster. Advanced processing methods are likely to mitigate the safety risks associated with injectable collagen products, though further research is needed to validate their biological effectiveness and clinical benefits.

## 1. Introduction

Collagen helps to maintain skin’s firmness, elasticity, and hydration. As we age, the body’s natural collagen production decreases, leading to the formation of wrinkles, sagging skin, and the loss of volume. Collagen treatments, such as oral collagen supplements or injectables, aim to replenish lost collagen and improve skin elasticity, hydration, and overall appearance [1].

Oral collagen supplements are available as powder, liquid, pills, and gummies and are sourced from bovine, porcine, marine, and other sources [2,3]. Injectables such as skin boosters are directly injected into the epidermis [4,5,6], and multiple studies/trials have demonstrated revitalization, hydration, and rejuvenation of the skin [4,5,6,7,8,9].

The global tissue-engineered collagen biomaterial market will be valued at over USD 6.63 billion by 2025 [10]. The market for oral collagen supplements surpassed USD 1 billion in 2022 and is projected to grow at more than 6.5% annually from 2023 to 2032. However, despite their widespread use for cosmetic purposes to improve skin functions, there is insufficient clinical evidence to support oral collagen supplements [11]. Although some clinical trials appear promising, it is still unclear if the skin improvements are due to oral collagen supplements.

On the other hand, the market for injectable skin boosters is already valued at USD 1.08 billion in 2023 and is expected to grow at a rate of 9.0% annually from 2024 to 2030 [12]. For injectable skin boosters to be considered effective, they must be safe, natural, have low downtime, be rapidly effective, and by consistently delivered via minimally invasive methods [13]. Overall, direct injection of skin boosters appears to be more beneficial/effective for skin improvements compared to oral collagen supplements.

Previously commercialized injectable collagens were aseptically processed by filtration in a clean bench or cleanroom to avoid collagen degradation due to sterilization limitations [14]. However, aseptic processing like the use of only filtration cannot provide the same quantitative level of sterility assurance as terminal sterilization [15]. Autoimmunity of injectable collagen has been reported in animal models and clinical studies [16,17,18]. It is therefore essential to establish clinically safe processing technologies for sterile collagen products whilst avoiding protein denaturation and undesirable modifications after processing and sterilization [19,20].

Despite the safety concerns, injectable collagen can mediate physiological effects during tissue repair [21]. Collagen products play an important role in maintaining tissue health and in the treatment of many conditions such as skin wound healing, gastroesophageal reflux, and conditions affecting the elderly such as skin regeneration, sarcopenia (muscle wasting), and bone loss [22]. Numerous clinical trials have shown that collagen-based therapies are effective [22]. Degraded proteins of the extracellular matrix generate fragments called matrikines that not only aid the pathology of skin diseases and aging but also provide potential mechanisms of repair [23]. The manufacturing process for animal-derived collagen typically involves several steps such as source selection, extraction, cleaning and washing, acid or enzymatic treatment, purification, concentration and drying, and sterilization [24]. However, there are some deficiencies and challenges associated with these processes, which can affect the collagen quality, increase the risk of contamination, and lead to degradation [25]. To mitigate all the safety concerns of injectable collagen, a consistent and verified manufacturing process is necessary to confirm a minimal immune response, consistent integrity after sterilization, viral inactivation, and a controlled degradation profile.

To address these requirements, we conducted a study on pre-filtered type I collagen that was purified with multiple filtration steps and post-sterilized by low-temperature steamed sterilization. We tested the sterility, cytotoxicity, collagen expression, viscosity pattern, degradation, and integrity in vitro and biocompatibility in vivo after intramuscular implantation tests in rats. The goal of this study was to confirm the biological safety of type I collagen after these improved purification and sterilization steps.

## 2. Results and Discussion

### 2.1. Sterility Test

We used a microbiology sterility test to confirm the sterility of the collagen gel by inoculating the test samples on Tryptic Soy Agar (TSA), which can detect aerobic microbes and fungus at 22.5 ± 2.5 °C, and Fluid Thioglycollate Medium (FTM), which can detect anaerobic microbes at 32.5 ± 2.5 °C (Figure S1). After culturing for 7 and 14 days, the cultured media were visually inspected for microbe or fungus colony formation. The microbiology sterility test results showed that none of the TSA and FTM media cultured with test samples or negative controls indicated the presence of any microbes after the culture (Table 1).

### 2.2. Cytotoxicity Test

**Figure 1:**
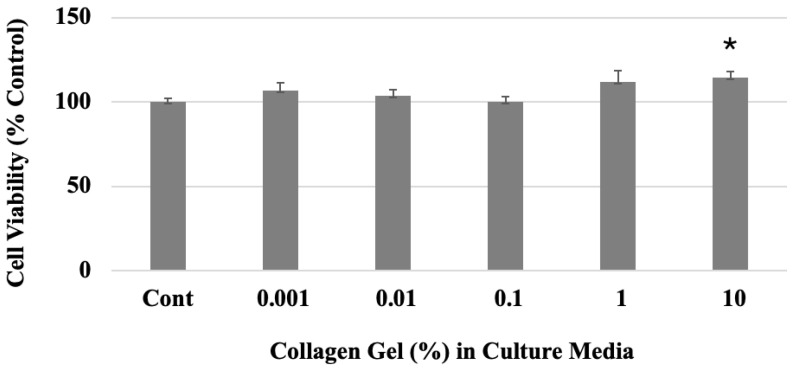
Cell viability of human dermal fibroblasts in the culture media containing 10% collagen gel (n = 3). * *p* < 0.05 (one-way ANOVA) as compared to control (Cont).

The MTT assay results showed no evidence of cytotoxicity following exposure of human dermal fibroblast (HDF) to a range of collagen concentrations (%*w*/*w*) in culture (Figure 1).

The culture media containing 10% collagen gel led to an increased cell viability (114.53%) of the HDFs. This suggests that the collagen gel did not affect the cytotoxicity, but had a positive cell proliferation effect as the gel concentration increased.

### 2.3. Collagen Expression

**Figure 2:**
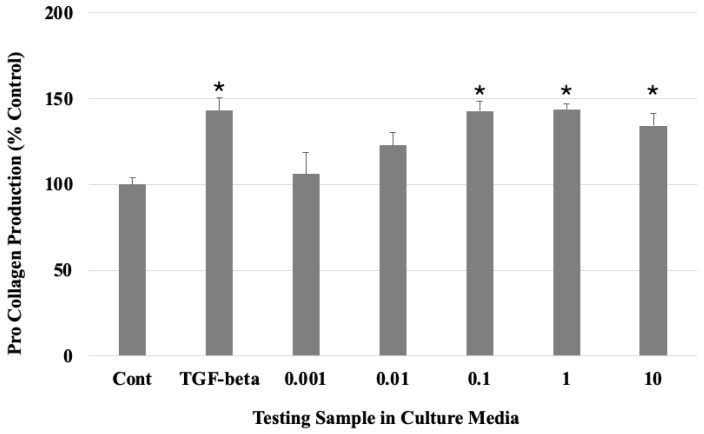
Procollagen expression of human dermal fibroblasts in the culture media containing different % collagen gel (n = 3). * *p* < 0.05 (one-way ANOVA) as compared to control (Cont).

The ELISA assay results showed that the HDFs produced procollagen 1α1 in the collagen gel mixed-culture media (Figure 2).

The 0.1% collagen gel culture media (142.86%), 1% collagen gel culture media (143.69%), and 10% collagen gel culture media (134.24%) produced more procollagen than the control group. The increase in these collagen gel testing groups showed a similar increase with the positive control of the TGF-beta (5 ng/mL) culture media treatment.

### 2.4. Gel Viscosity

**Figure 3:**
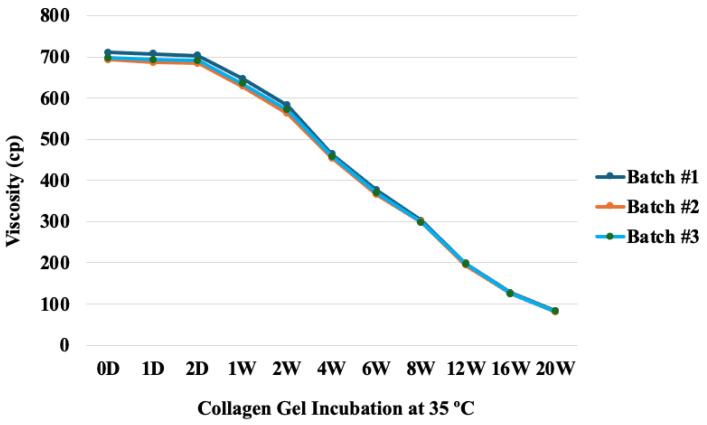
Collagen gel viscosity (n = 3) at different time points (days and weeks).

The initial viscosity (700.11 cP) of the collagen gel at 35 °C was maintained by day+2, and the gel viscosity gradually decreased over 20 weeks (Figure 3).

The half-life of the viscosity was between 6–7 weeks. The decrease in the gel viscosity was noted by 1 week and decreased 7-fold by 20 weeks. The linear trend from 2 weeks to 20 weeks indicated that the viscosity would be zero between 22 weeks and 23 weeks. The viscosity patterns at the different time points showed batch consistency after the processing and sterilization.

### 2.5. SDS-PAGE Gel Electrophoresis

**Figure 4:**
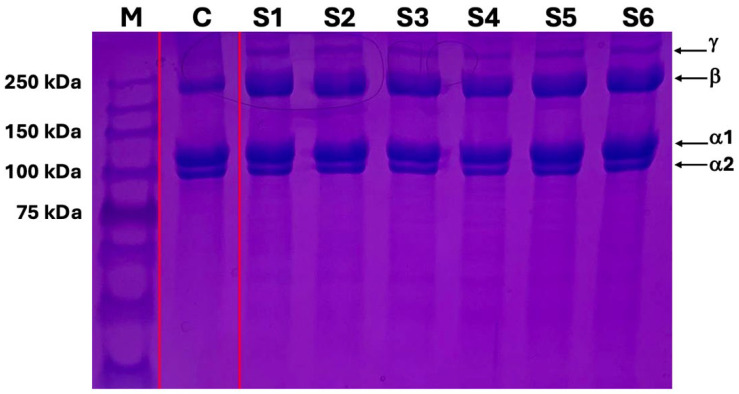
SDS-PAGE gel electrophoresis of type I collagen gel: M; protein marker, C; control, commercial type I collagen gel, S1–S6; filtered and sterilized type I collagen gel (6 different batches), γ and β; collagen secondary structure of gamma- (over 250 kDa) and beta- (240–250 kDa)bands, α1 and α2; the basic structure of monomeric alpha-1 and alpha-2 monomers (100–130 kDa).

SDS-PAGE gel electrophoresis showed that the bands of the terminally sterilized collagen gel from 6 different batches were the same as the control material, type I collagen gel. Moreover, there were no smeared bands and smaller-sized protein fragments on the gel, suggesting that the terminally filtered and sterilized collagen gel did not induce collagen degradation nor polypeptide chain modification (Figure 4).

### 2.6. Microscopic Evaluations

**Figure 5:**
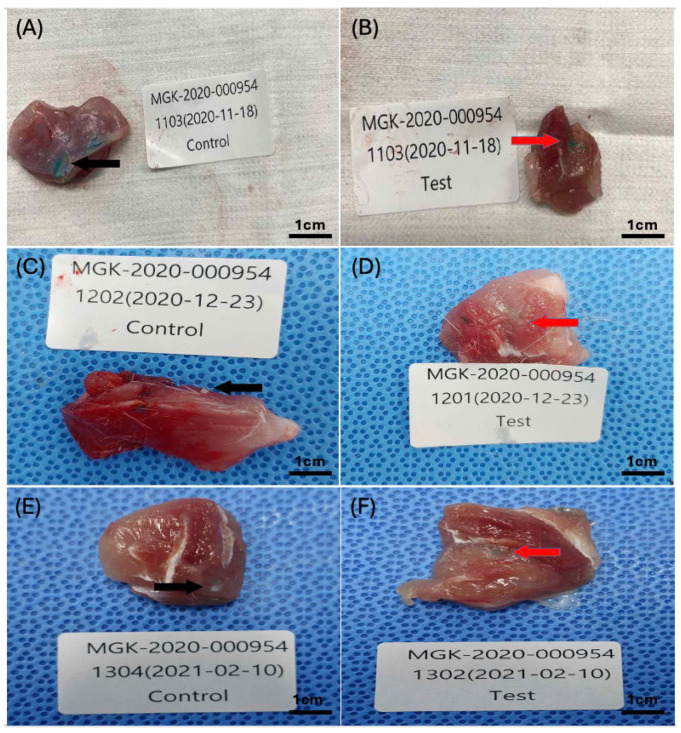
Collected tissue specimens from implantation sites after implantation periods of 1, 6, or 13 weeks: (**A**) control-sample-implanted tissue specimen after 1 week (n = 11), (**B**) test-sample-implanted tissue specimen after 1 week (n = 12), (**C**) control-sample-implanted tissue specimen after 6 weeks (n = 10), (**D**) test-sample-implanted tissue specimen after 6 weeks (n = 12), (**E**) control-sample-implanted tissue specimen after 13 weeks (n = 11), (**F**) test-sample-implanted tissue specimen after 13 weeks (n = 10). Black arrows on (**A**,**C**,**E**) indicate the implanted control sample’s location on the collected tissue specimens, and red arrows on (**B**,**D**,**F**) indicate the implanted test sample’s location on the collected tissue specimens.

A macroscopic lesion was observed after the excision of the implantation site with the adjacent tissue (Figure 5).

**Figure 6:**
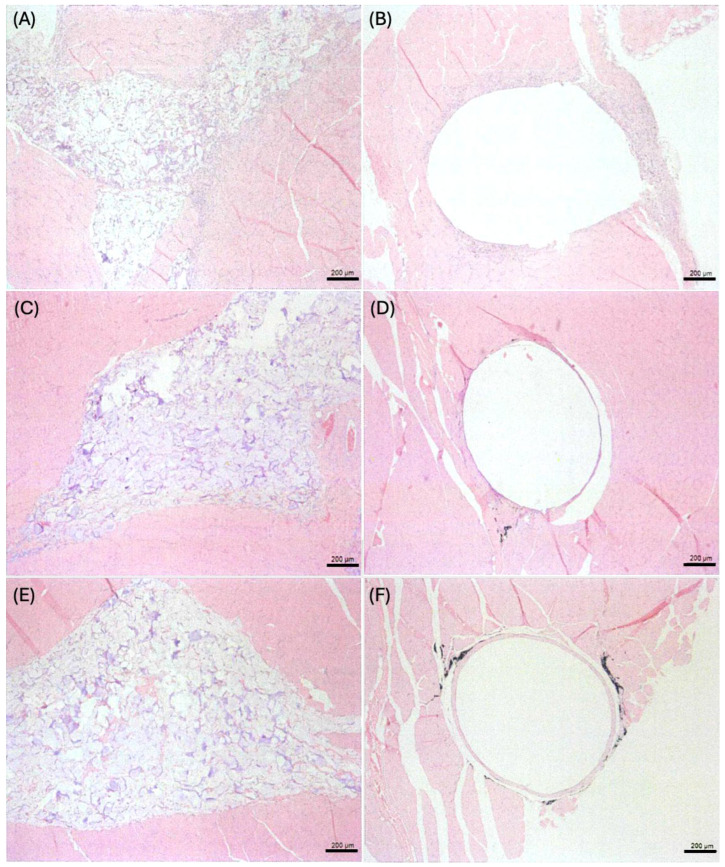
H&E staining of muscle sections at the implantation site: (**A**) test-sample-implanted tissue after 1 week, (**B**) control-sample-implanted tissue after 1 week, (**C**) test-sample-implanted tissue after 6 weeks, (**D**) control-sample-implanted tissue after 6 weeks, (**E**) test-sample-implanted tissue after 13 weeks, (**F**) control-sample-implanted tissue after 13 weeks.

After 1 week of implantation, polymorphonuclear leukocytes, lymphocytes, and macrophage infiltration were observed in the implantation sites for both the test and control samples (Figure 6A,B). After 6 and 13 weeks of implantation, polymorphonuclear leukocytes, lymphocytes, and macrophage infiltration were reduced compared to the samples after 1 week of implantation (Figure 6C–F).

**Figure 7:**
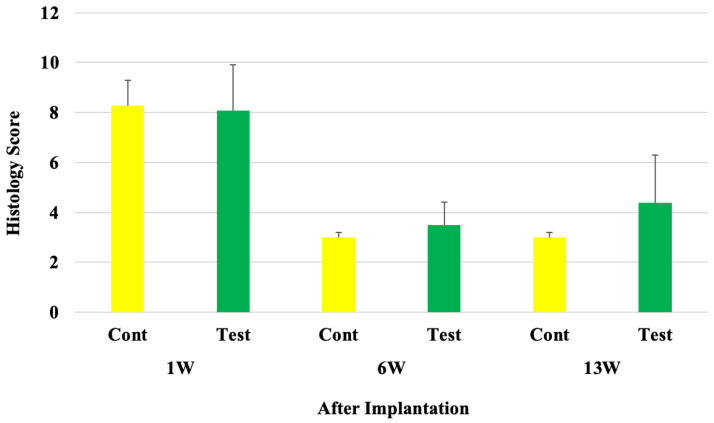
Microscopic evaluation of histology samples: 1, 6, and 13 weeks after implantation: Cont: control-sample-implanted rat group (n = 10–12), Test: test-sample-implanted rat group (n = 10–12).

After 1 week of implantation, the average microscopic evaluation score was 8.3 in the control group and 8.1 in the test group. After 6 weeks of implantation, the average microscopic evaluation score was reduced to 3.0 in the control group and 3.5 in the test group. After 13 weeks of implantation, the average score was 3.0 in the control group and 4.4 in the test group (Figure 7).

One week after implantation, there was no significant difference between the groups. The histopathological evaluation index for the test sample was evaluated as 0.0 (Test average−Control average) compared with the control group. After 6 weeks, no significant difference between the groups was observed. The histopathological evaluation index for the test sample was evaluated as 0.5 compared with the control group, and after 13 weeks, no significant difference between the groups was noted. The histopathological evaluation index for the test sample was evaluated as 1.4 compared with the control group. Therefore, for all time points tested after implantation, minimal or no reaction in terms of the bio-reactivity rating was seen (Table 2).

A skin booster is an injectable treatment used to restore and improve the overall quality and health of the skin [4]. However, it can cause side effects such as swelling, redness, bruising, and pain at the injected site. A major factor leading to side effects is the reaction to ingredients, making it important to validate the process used for hydrogel production before clinical use [26].

Improved sterilization of sensitive biomaterials is technically limited; however, it is a very important area to explore [27]. To ensure the safe use of type I collagen gel as a skin booster, it must undergo verified processing technique(s) and standard testing for sterility, adverse reactions, and stability. In this paper, we propose a new processing technique, which involves a double-filtration process using filters with different cut-off sizes and low-temperature steam sterilization. The verification of the process technique is very critical because degraded or modified collagen by post-processing steps may have potential safety risks such as autoimmunity, allergic reactions, inflammation, and impaired functionality on the skin [28].

Other studies have reported that post-processing and sterilization can degrade collagen [29,30]. First, we confirmed the sterility of the post-processed collagen gel with a sterility test. We performed cytotoxicity and new collagen expression tests by co-culturing of human fibroblasts and collagen gel mixed-culture media. The post-processed collagen gel had no cytotoxicity but showed a cell proliferation effect as we increased the collagen concentration of the culture media. The human fibroblasts cultured in the collagen gel mixed-culture media produced new procollagen with a similar level to the cultured cells in the TGF-beta media. This also suggests that the collagen gel enhanced cell proliferation and promoted the cells to increase the production of new procollagen. The consistency of gel viscosity from different batches demonstrated the physical stability and biological compatibility of the gel after the processing and sterilization. To assess the integrity of the collagen gel after processing and sterilization in vitro, we performed SDS-PAGE gel electrophoresis to analyze the protein bands. The protein gel electrophoresis results showed that the type I collagen gel maintained the basic structure of monomeric alpha-1, alpha-2, beta, and gamma chains compared to the other commercially available type I collagen gel. The results of the SDS-PAGE gel electrophoresis confirmed the maintenance of the collagen integrity after our processing and sterilization methods.

To evaluate the local effects of the purified and low-temperature-sterilized collagen gel in vivo, the collagen gel was implanted in the intramuscular tissue of rats and evaluated at 1 week, 6 weeks, and 13 weeks after implantation. On histologic evaluation, there was no significant increase in polymorphonuclear leukocyte infiltration, lymphocyte infiltration, macrophage infiltration, neovascularization, and fibrosis at the test implantation sites relative to the control. The histopathological evaluation index was 0.0 after 1 week, 0.5 after 6 weeks, and 1.4 after 13 weeks of implantation.

The in vivo evaluations also supported that the implanted collagen gel in the intramuscular tissue of rats did not show any critical tissue reactions and confirmed its biological safety. However, other collagen-related studies have shown tissue inflammation and immune reactions using irradiated, chemically processed, or high-temperature-sterilized methods typically employed for other collagens [31,32]. Based on the collagen integrity, sterility, and animal study for biological safety evaluation, this study’s findings support that the implanted collagen gel produced using double-filtration and low-temperature sterilization processing methods demonstrates favorable outcomes, and the methods are effective and safe for potential clinical applications.

The demonstration of the collagen gel product’s relative biological effectiveness requires further investigation with an animal model. Future studies such as full-thickness wound healing and assessments of sub-chronic systemic toxicity will evaluate the efficacy of treating soft tissue defects in appropriate animal models.

## 3. Conclusions

This study aimed to examine the sterility, cytotoxicity, new collagen production by cell proliferation, viscosity, protein integrity, and animal histopathology of pre-filtered and low-temperature-sterilized collagen gels in both in vitro and in vivo experiments. Based on these results, the collagen showed sterility, no cytotoxicity, enhanced cell proliferation, and a consistent gel viscosity. The collagen after processing and sterilization maintained its integrity without any protein denaturation and modification in vitro, and the bioactivity rating of the implanted test sample was considered non-irritant in the in vivo rat model. Therefore, purified and low-temperature-sterilized type I collagen gel is a promising candidate as a safe and injectable collagen gel product for clinical use.

## 4. Materials and Methods

### 4.1. Preparation of Test Samples

Collagen (D-med, Seoul, Republic of Korea) was extracted from porcine skin as described earlier [33]. The extracted collagen gel was filtered sequentially using a primary filter (Polysep II Cartridge, MilliporeSigma, Burlington, MA, USA) with a 300 kDa cut-off size and a secondary filter with a 100 kDa cut-off size (Milligard Cartridge, MilliporeSigma, Burlington, MA, USA). The final filtered collagen gel (3% *w*/*v*, 150 kDa) was then sterilized at 40 °C and 0.7 atm for 40 min in a steam sterilization chamber (HS-1000R, Hanshinmed, Seoul, Republic of Korea). The collagen gel for this study was prepared in a 1 mL syringe. A high-density polyethylene rod (diameter: 1 mm, length: 5 mm) was sterilized at 121 °C for 15 min and used as a control sample.

### 4.2. Preparation of Sterility Test

To confirm the sterility of the test sample, Tryptic Soy Agar (TSA, Synergyinno, Seongnam, Republic of Korea) and Fluid Thioglycollate Medium (FTM, Synergyinno, Seongnam, Republic of Korea) were used for the microbiology sterility testing according to the USP 29<71> test method [34]. As a positive control for the sterility testing, *Staphylococcus aureus* (ATCC 6538, ThermoFisher Scientific, Seoul, Republic of Korea), *Pseudomonas aeruginosa* (ATCC 9027, ThermoFisher Scientific, Seoul, Republic of Korea), *Bacillus subtilis* (ATCC 6633, ThermoFisher Scientific, Seoul, Republic of Korea), *Candida albicans* (ATCC 10231, ThermoFisher Scientific, Seoul, Republic of Korea), and *Aspergillus niger* (ATCC 16404, ThermoFisher Scientific, Seoul, Republic of Korea) were used during the culture.

### 4.3. Cell Culture and Cytotoxicity Test

Human dermal fibroblasts (HDFs, PCS-201-012, ATCC, Manassas, VA, USA) were cultured in Dulbecco’s modified Eagle’s medium (DMEM) including 10% fetal bovine serum (FBS) and 1% penicillin streptomycin (PS). HDFs were plated on a 48-well plate at a 5 × 10^4^ cells/mL density, and the cells were incubated at 37 °C in 5% CO_2_ for 24 h. After incubation, the culture media were aspirated, replaced with serum-free media mixed with different volumes of collagen gels (0.001, 0.01, 0.1, 1, and 10% *v*/*v*), and incubated for 24 h. After incubation, the serum-free media were aspirated, replaced with 1 µg/mL concentrated 3-(4,5-dimethythiazol-2-yl)-2,5-diphenyl tetrazolium bromide (MTT, Sigma-Aldrich, St. Louis, MO, USA) media, and incubated for 3 h. The MTT media were removed, and 100 µL of dimethyl sulfoxide (DMSO) was added to each well. The color changes due to the conversion of MTT to formazan dye were measured using a microplate reader at a wavelength of 540 nm.

### 4.4. Quantitative Analysis of Collagen Expression

HDFs were plated on a 48-well plate at a 5 × 10^4^ cells/mL density, and the cells were incubated at 37 °C in 5% CO_2_ for 24 h. After incubation, the culture media were aspirated, replaced with serum-free media mixed with different volumes of collagen gels (0.001, 0.01, 0.1, 1, and 10% *v*/*v*), and incubated for 24 h. A negative control was used that comprised HDFs cultured in serum-free media only, and a positive control was used that comprised HDFs cultured in serum-free media mixed with TGF-beta (5 ng/mL). The supernatant per well was collected and quantified with an ELISA assay kit (Human procollagen 1α1, R&D systems, Seoul, Republic of Korea) according to the manufacturer’s guidelines.

### 4.5. Viscosity Analysis of Collagen Gels

Post-processed and sterilized collagen gels from 3 different batches were pre-filled into a 10 cc syringe. The syringes pre-filled with collagen gel were incubated at 35 °C, and at each time point (days 1 and 2, weeks 1, 2, 4, 6, 8, 12, 16, and 20), the syringes stayed at ambient temperature for 2 h before measuring the viscosity with a viscometer (DVII Pro, Brookfield, Toronto, ON, Canada). A total of 0.5 mL of collagen gel was collected from the syringe, and the viscosity was measured at a 20~25 °C target temperature and a 2.0 N s^−1^ shear rate. The gel’s viscosity was measured for 11 min with the viscometer, and the data from the viscometer were collected 10 times, once per minute.

### 4.6. SDS-PAGE Analysis of Processed and Sterilized Collagen Gel

The test collagen samples (n = 6) were diluted to 1 mg/mL with deionized (DI) water. The diluted collagen was mixed with 5× loading buffer (#426311, BioLegend^®^, Seoul, Republic of Korea) and boiled at 95 °C for 5 min. The samples were separated by 5%–7% resolving gel and stained with 0.05% Coomassie brilliant blue (B0149-25G, Sigma-Aldrich, St. Louis, MO, USA) for an hour and destained with a destaining solution for 20 min and DI water for 30 min. The protein ladder (HQM14500, BioD, Seoul, Republic of Korea) was used to evaluate the molecular weights of the type I collagen. After the destaining, the gel was scanned by a gel scanner (Bio-6000, BIOIMAGER, Seoul, Republic of Korea). The molecular weight of the stained gel bands was identified by comparing them with the protein ladder.

### 4.7. Rat Muscle Implantation Model

This animal study was approved by the institutional animal care of use committee of the Korea Testing and Research Institute (IAC202846). A total of 18 rats (Sprague Dawley^®^ specific-pathogen-free rats, 18 males, 9 weeks old, Orient Bio Co., Ltd., Seongnam, Republic of Korea) were used for muscle implantation under GLP (Good Laboratory Practice) and per the ISO10993-6:2016 standard [35,36]. For each implantation group of 1, 6, and 13 weeks, 6 rats were used for each time point. The viscosity analysis confirmed that the test samples maintained their gel viscosity characteristics at the selected time points. Each rat had 2 test implantation sites and 2 control implantation sites, leading to a total of 12 test implantation and 12 control implantation sites per time point.

**Figure 8:**
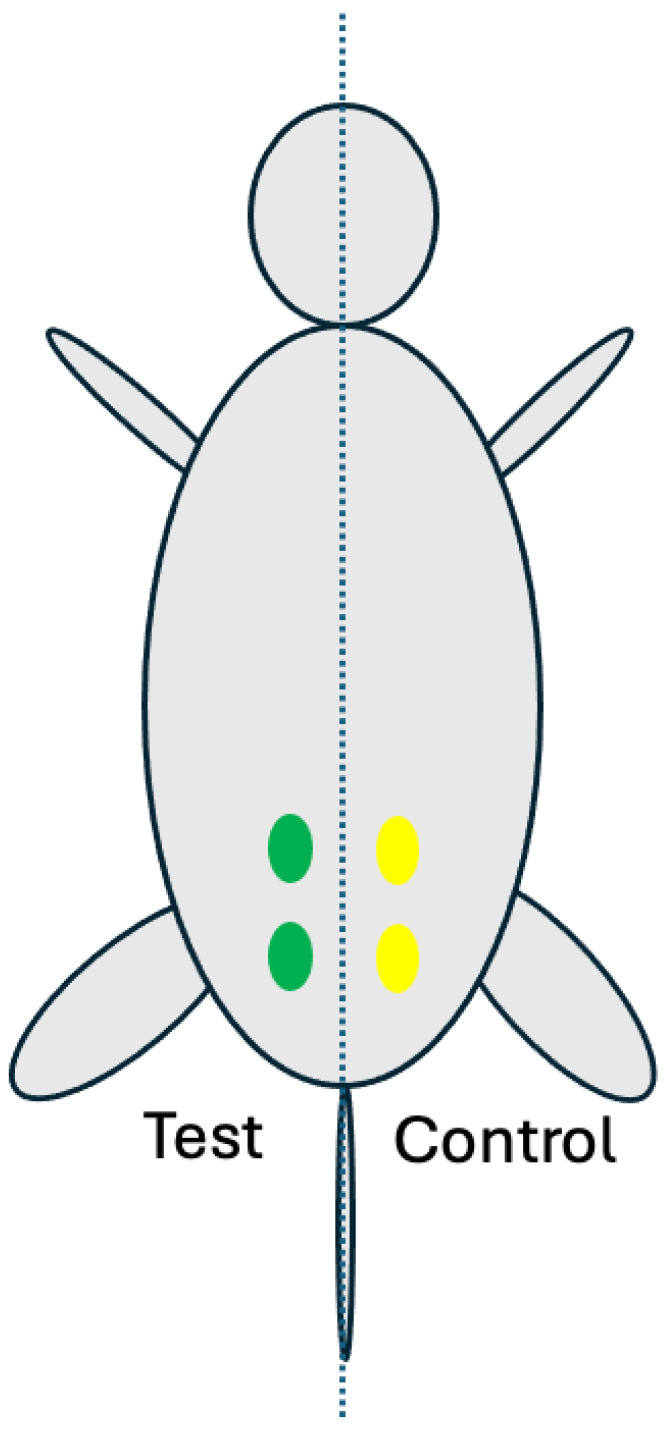
Implantation locations (four) on the gluteal muscle of a rat: the left two green marks are implantation sites for two test samples and the right two yellow marks are implantation sites for two control samples.

Rompun (xylazine, Bayer, Republic of Korea) and ketamine (ketamine, Yuhan, Republic of Korea) were injected intraperitoneally for anesthesia. After inducing anesthesia, the fur over the gluteal muscle area was removed using a clipper, and the implantation area was disinfected with betadine. The skin was incised to expose the gluteal muscle. For each animal, two test samples (0.2 mL each) were implanted in the left gluteal muscle and two control samples (0.2 mL each) were implanted in the right gluteal muscle (Figure 8).

After recovery from anesthesia, the animals were placed in a cage. All animals were observed daily for nonspecific clinical signs such as anorexia, lethargy, emaciation, ruffled fur, and acute death. At the appropriate time points, the animals were euthanized by carbon dioxide.

After full fixation in 10% neutral buffered formalin for up to 3 days, the tissue specimens were embedded in paraffin for hematoxylin and eosin (H&E) staining and histopathological examination. Tissue specimens were trimmed into 10 um pieces by using a rotary microtome (RM2125, Leica Biosystems, Seoul, Republic of Korea) and then cut into 4 µm sections. The histology sections were immersed in water, stained with H&E, and mounted. The stained slides were examined under an inverted microscope at a magnification of 50×.

### 4.8. Microscopic Evaluation

The histological evaluation of the implantation sites was evaluated using a previously described method [33] outlined in Table 3 and Table 4. The mean scores by double-blind evaluation were averaged from 10–12 histology samples per group at each time point. The total score of biological response was divided into the number of implantation sites.

After calculating the difference in the mean score between the test sample and the control sample, the bio-reactivity rating was evaluated (Table 4).

## Figures and Tables

**Table 1:** Sterility test of the collagen gel at D-0, D+7, and D+14.

Media	Test Samples	D-0	D+7	D+14
TSA	#01	No growth	No growth	No growth
	#02	No growth	No growth	No growth
	#03	No growth	No growth	No growth
	Negative control	No growth	No growth	No growth
	Positive control	No growth	Growth	Growth
Positive control in TSA: *Staphylococcus aureus*, *Pseudomonas aeruginosa*, and *Clostridium sporogenes*				
FTM	**Test Samples**	**D-0**	**D+7**	**D+14**
	#01	No growth	No growth	No growth
	#02	No growth	No growth	No growth
	#03	No growth	No growth	No growth
	Negative control	No growth	No growth	No growth
	Positive control	No growth	Growth	Growth
Positive control in FTM: *Bacillus subtilis*, *Candida albicans*, and *Aspergillus niger*				

**Table 2:** Bioactivity rating after 1 week, 6 weeks, and 13 weeks of implantation.

Implantation Period	Mean Score (Control)	Mean Score (Test)	Mean Score Difference (Test−Control)	Bio-Reactivity Rating
1 week	8.3	8.1	8.1 − 8.3 = −0.2 (Considered as 0.0)	Minimal or No Reaction
6 weeks	3.0	3.5	3.5 − 3.0 = 0.5	Minimal or No Reaction
13 weeks	3.0	4.4	4.4 − 3.0 = 1.4	Minimal or No Reaction

**Table 3:** Histological evaluation system.

Category	Score				
	0	1	2	3	4
Leukocyte	0	Rare, 1–5/phf *	5–10/phf	Heavy infiltrate	Packed
Lymphocytes	0	Rare, 1–5/phf	5–10/phf	Heavy infiltrate	Packed
Macrophages	0	Rare, 1–5/phf	5–10/phf	Heavy infiltrate	Packed
Neo-vascularization	0	Minimal capillary proliferation, focal, 1–3 buds	Groups of 4–7 capillaries with supporting fibroblastic structures	Broad band of capillaries with supporting structures	Extensive band of capillaries with supporting fibroblastic structures
Fibrosis	0	Narrow band	Moderately thick band	Thick band	Extensive band

* phf: per high-powered (50×) field.

**Table 4:** Bio-reactivity rating.

Bio-Reactivity Rating	Mean Score (Animal Score)
Minimal or no reaction	0.0–2.9
Slight reaction	3.0–8.9
Moderate reaction	9.0–15.0
Severe reaction	>15

## Data Availability

The original contributions presented in the study are included in the article/supplementary material, further inquiries can be directed to the corresponding authors.

## Supplementary Materials

The following supporting information can be downloaded at: https://www.mdpi.com/article/10.3390/gels10100619/s1, Figure S1: The collagen gels were tested for confirming the sterility by inoculating the test samples on Tryptic Soy Agar (TSA) at 22.5 °C and Fluid Thioglycollate Medium (FTM) at 32.5 °C and culturing for 7 and 14 days. TSA can detect aerobic microbes and fungi at 22.5 °C, and FTM can detect anaerobic microbes at 32.5 °C. The growth of microbes showed any turbidity in the culture media (see red arrows).
