# Supplementary material for: Biocompatibility Study of Purified and Low-Temperature-Sterilized Injectable Collagen for Soft Tissue Repair: Intramuscular Implantation in Rats
Source: Gels. 2024 Sep 26;10(10):619. doi: 10.3390/gels10100619 (PMC11508103; doi:10.3390/gels10100619)
Supplement: Supplementary file 1 [file gels-10-00619-s001.zip › gels-3136279-supplementary.pdf]

gels

Article

Biocompatibility Study of Purified and Low-Temperature-Sterilized Injectable Collagen for Soft Tissue Repair: Intramuscular Implantation in Rats

Tae-Hoon Koo [1], Jason K. Lee [2,*], Shawn P. Grogan [2], Ho Jong Ra [2] and Darryl D. D'Lima [2,*]

[1] D.med LLC, 111, Sagimakgol-ro, Jungwon-gu, Seongnam-si, Gyeonggi-do, 13202, Republic of Korea; glenkoo@dmed.com (T.H.K.)
[2] Shiley Center for Orthopaedic Research and Education at Scripps Clinic, 10666 N Torrey Pines Road, MS126, La Jolla, CA 92037, USA; sgrogan@scripps.edu (S.P.G.)
* Correspondence: jason.lee@scripps.edu (J.K.L.); ddlima@scripps.edu (D.D.D.); Tel.: +1-858-926-9808 (J.K.L.); +1-858-784-9036 (D.D.D.)

Citation: Koo, T.-H.; Lee, J.K.; Grogan, S.P.; Ra, H.J.; D'Lima, D.D. Biocompatibility Study of Purified and Low-Temperature-Sterilized Injectable Collagen for Soft Tissue Repair: Intramuscular Implantation in Rats. *Gels* **2024**, *10*, 619. https://doi.org/10.3390/gels10100619

Academic Editor: Rodney Dilley

Received: 15 August 2024
Revised: 13 September 2024
Accepted: 24 September 2024
Published: 26 September 2024

Copyright: © 2024 by the authors. Licensee MDPI, Basel, Switzerland. This article is an open access article distributed under the terms and conditions of the Creative Commons Attribution (CC BY) license (https://creativecommons.org/licenses/by/4.0/).

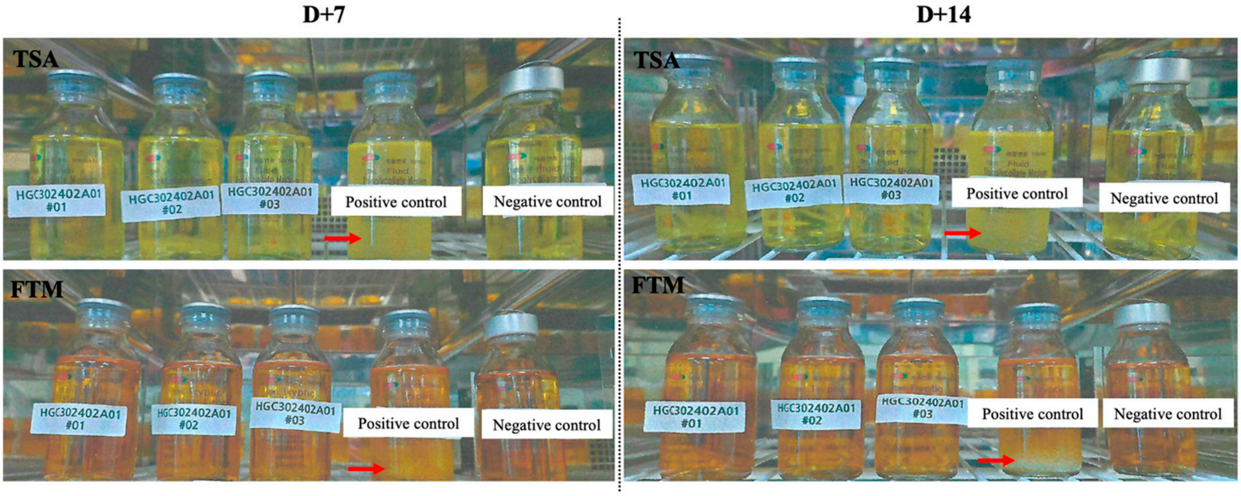

Figure S1. Tryptic Soy Agar (TSA) at 22.5 °C, and Fluid Thioglycollate Medium (FTM) at 32.5 °C were inoculated with the collagen gel test samples and cultured for 7 and 14 days. TSA can detect aerobic microbes and fungi at 22.5 °C, and FTM can detect anaerobic microbes at 32.5 °C. The growth of microbes shows any turbidity in the culture media (see red arrows).
